# Extracellular secretion of a recombinant therapeutic peptide by *Bacillus halodurans *utilizing a modified flagellin type III secretion system

**DOI:** 10.1186/1475-2859-10-62

**Published:** 2011-08-04

**Authors:** Eldie Berger, Michael C Crampton, Nolwandle P Nxumalo, Maureen E Louw

**Affiliations:** 1 CSIR Biosciences, Box 395, Pretoria 0001, South Africa

## Abstract

**Background:**

Through modification of the flagellin type III secretion pathway of *Bacillus halodurans *heterologous peptides could be secreted into the medium as flagellin fusion monomers. The stability of the secreted monomers was significantly enhanced through gene-targeted inactivation of host cell extracellular proteases. In evaluating the biotechnological potential of this extracellular secretion system an anti-viral therapeutic peptide, Enfuvirtide, was chosen. Currently, Enfuvirtide is synthesised utilizing 106 chemical steps. We used Enfuvirtide as a model system in an effort to develop a more cost-effective biological process for therapeutic peptide production.

**Results:**

An attempt was made to increase the levels of the fusion peptide by two strategies, namely strain improvement through gene-targeted knock-outs, as well as vector and cassette optimization. Both approaches proved to be successful. Through chromosomal inactivation of the *spo0A, lytC *and *lytE *genes, giving rise to strain *B. halodurans *BhFDL05S, the secretion of recombinant peptide fusions was increased 10-fold. Cassette optimization, incorporating an expression vector pNW33N and the N- and C-terminal regions of the flagellin monomer as an in-frame peptide fusion, resulted in a further 3.5-fold increase in the secretion of recombinant peptide fusions.

**Conclusions:**

The type III flagellar secretion system of *B. halodurans *has been shown to successfully secrete a therapeutic peptide as a heterologous flagellin fusion. Improvements to both the strain and expression cassette led to increased levels of recombinant peptide, showing promise for a biotechnological application.

## Background

Certain classes of pathogenic bacteria secrete virulence proteins in a Sec-independent manner by means of a type III secretion system (TTSS). Based on homologous component proteins with common physico-chemical properties the flagellin system was included as part of the TTSS [1]. A feature of the TTSS that differentiates it from other secretion systems is the lack of a defined amino acid secretion signal to direct substrate proteins for secretion. In addition, secretion occurs either across the cell membranes or the cell wall through a channel [2]. Most flagellar components are translocated across the cytoplasmic membrane by the flagellar type III system where they self- assemble at the distal end of the flagellin channel with the help of a cap structure. Flagellar proteins synthesized in the cytoplasm are targeted to the export apparatus with the help of flagellum-specific chaperones. The flagellin protein is then pushed into the channel by an ATPase, whose activity is controlled by its regulator to enable the energy of ATP hydrolysis to be efficiently coupled to the translocation reaction [3]. The flagellar type III secretion apparatus was found to efficiently transport the flagellin subunit protein FliC in both Gram-positive and Gram-negative bacteria. It has been shown that *Escherichia coli *mutants with a non-functional FliD protein (cap structure) fail to assemble flagella and FliC monomers diffuse into the culture medium [4]. Moreover, it was shown that an *E. coli *mutant (defective in both the *fliC *and *fliD *genes) was able to secrete heterologous polypeptide fusions into the growth medium. This was facilitated by fusing a 173-bp untranslated DNA fragment upstream of the *fliC *gene, as well as a transcriptional terminator from *fliC *downstream of the gene encoding the polypeptide of interest [4]. In a related approach, harnessing the *Salmonella *type III flagellin pathway, Végh et al [5] used a *Salmonella *strain defective in flagellin gene expression, but still apparently possessing a functional *fliD *gene to show heterologous secretion of polypeptide-FliC fusions. They demonstrated that a 22-residue long segment within the disordered N-terminal region of *Salmonella *flagellin contains the recognition signal for the flagellar export machinery. Dobó et al [6] evaluated the secretion of a number of different polypeptides using this system. They found that the nature of the attached polypeptide had a significant effect on secretion efficiency.

*Bacillus halodurans *Alk36 was found to over-produce flagellin protein and this ability was utilized for the development of a surface display system [7]. Berger et al [8] demonstrated that when the flagellin type III secretion pathway of this Gram-positive *Bacillus *strain was modified by the inactivation of both the *hag *gene (encoding FliC protein) and *fliD *gene (encoding the FliD protein), then heterologous peptides could be secreted into the medium as flagellin fusion monomers. The gene encoding the human immunodeficiency virus (HIV) subtype C antigenic peptide was fused as an in-frame sandwich fusion into the central variable region of the *hag *gene. The stability of the secreted flagellin fusion monomers was shown to be significantly enhanced through gene-targeted inactivation of five proteases.

In evaluating the biotechnological potential of this *B. halodurans *expression system, the anti-viral therapeutic peptide Enfuvirtide, currently marketed by Roche under the trademark Fuzeon^®^, was expressed. It is a 36-amino acid non-glycosylated synthetic peptide that blocks HIV infection by preventing the virus from entering the cell. It is currently synthesised utilizing 106 chemical steps. The peptide is modified by acetylation at the N-terminus and amidation at the C-terminus, which enhances stability and efficiency of the peptide. This complicated manufacturing process contributes towards the high cost of the drug [9]. Therefore there is a clear need to develop a cost-effective fermentation-based process for recombinant therapeutic peptide production.

In this study, we report further refinements to the *B. halodurans *host strain and expression cassette with a view to improving expression levels of a therapeutic peptide utilizing Enfuvirtide (Fuzeon^®^) as a model system. The Enfuvirtide peptide produced using a biological route, by recombinant gene expression, would, however, still need to be chemically modified for it to be regarded as a biosimilar.

## Methods

### Bacterial strains, plasmids and growth conditions

The bacterial strains and plasmids used in this study are listed in Table 1. Growth conditions, DNA techniques and *B. halodurans *transformation are described in Crampton et al [7]. Recombinant *B. halodurans *strains containing different constructs were grown in 50 ml of Luria Bertani (LB) broth (pH 8.5, chloramphenicol 10 μg/ml; 30°C on an orbital shaker at 175 rpm).

**Table 1:** Bacterial strains and plasmids used in this study

Strain or plasmid	Relevant characteristics	Source or reference
**Strains**		
*B. halodurans *BhFD05	Δ*hag*, Δ*wprA*, Δ*fliD*, Δ*alp*, Δ*apr*, Δ*vpr*, Δ*asp*	Berger et al (2009)
*B. halodurans *BhFDL05S	Δ*hag*, Δ*wprA*, Δ*fliD*, Δ*alp*, Δ*apr*, Δ*vpr*, Δ*asp*, Δs*po0A, ΔlytCE*	This study
*B. halodurans *BhFDL05SC	Δ*hag*, Δ*wprA*, Δ*fliD*, Δ*alp*, Δ*apr*, Δ*vpr*, Δ*asp*, Δs*po0A, ΔlytCE, ΔclpP*	This study
*B. halodurans *BhFDL05SM	Δ*hag*, Δ*wprA*, Δ*fliD*, Δ*alp*, Δ*apr*, Δ*vpr*, Δ*asp*, Δs*po0A, ΔlytCE, ΔflgM*	This study
*B. halodurans *BhFDL06S	Δ*hag*, Δ*wprA*, Δ*fliD*, Δ*alp*, Δ*apr*, Δ*vpr*, Δ*asp*, Δs*po0A, ΔlytCE*, Δ*aprX*	This study
*E. coli *DH10B	(F^- ^*mcr*A Δ(*mrr*-*hsd*RMS-*mcr*BC) (^φ^80d*lac*ZΔM15)Δ*lac*X74*end*A1 *rec*A1 *deo*R Δ(*ara-leu*) 7697 *ara*D139 *gal*U *gal*K *nup*G *rps*Lλ^- ^	Invitrogen
**Plasmids**		
pSEC194	Cm^R^, Ap^R^, thermosensitive integration vector	Crampton et al (2007)
pNW33N	*E. coli/Bacillus/Geobacillus *shuttle vector	Bacillus Genetics Stock Centre, (BGSC) Ohio, USA
pSECFliCFFuz	pSEC194, σ^D ^promoter, *hag *gene (FliC_1-275_), FLAG tag, enterokinase cleavage site and Enfuvirtide gene as in-frame fusions	This study
pNWFliCFFuz	pNW33N, σ^D ^promoter, *hag *gene (FliC_1-275_), FLAG tag, enterokinase cleavage site and Enfuvirtide gene as in-frame fusions	This study
pNWNCFFuz	pNW33N, σ^D ^promoter, truncated *hag *gene (FliC_Δ46-201_), FLAG tag, enterokinase cleavage site and Enfuvirtide gene as in-frame fusions	This study
pNWNFFuz	pNW33N, σ^D ^promoter, N- terminal *hag *gene (FliC_1-45_), FLAG tag, enterokinase cleavage site and Enfuvirtide gene as in-frame fusions	This study
pNWCFFuz	pNW33N, σ^D ^promoter, ATG, C-terminal *hag *gene (FliC_202-275_), FLAG tag, enterokinase cleavage site and Enfuvirtide gene as in-frame fusions	This study

### Gene targeted inactivation of the *spo0A, clpP, flgM *and *aprX *genes and the *lytCE *operon

Construction of mutant strains through gene-targeted inactivation was as described in Berger et al [8]. Essentially, this was achieved by creating a defective copy of the gene of interest through PCR amplification of two fragments containing part of the 5' and 3' regions of the gene using chromosomal DNA from *B. halodurans *as template. These fragments were ligated to the temperature sensitive vector pSEC194 transformed into the appropriate *B. halodurans *strain and the targeted gene partially deleted through a double-crossover event. Deletions were confirmed through PCR analysis (results not shown). The primers used to make these constructs are listed in Table 2. The primers were designed based on the nucleotide sequence of the relevant genes from the genome sequence for *B. halodurans *C125 available in the DNA Data Bank of Japan (http://www.ddbj.nig.ac.jp/) and is as follows: *spo0A *(BH2773), *clpP *(BH1930), *flgM *(BH3632), *aprX *(BH1930) and the *lytCE *operon (BH2665 and BH3671).

**Table 2:** List of primers, their corresponding nucleotide sequences and application

Primer name	Nucleotide sequence^a^	Application
SpoIVF	CGGAAAGCTTGTTGGTGCAGTGAC	Spo0A
SUpR	CTAGGATCCGTAAGAGGCTCCTAAATCAACCG	Spo0A
SDoF	CTAGGATCCGCCATGGTAGCCGATAAGC	Spo0A
SDoR	CTACCCGGGGATTAATCTGGGCAATGGTTGC	Spo0A
UplytCF	CTCGGTACCCTTAGAGGAGGGATGAGG	LytCE
UplytCR	CTCGGATCCACGGATGACGGTCACCAA	LytCE
DolytEF	CTCGGATCCTTGAACAAAGCCGCTGCA	LytCE
DolytER	CTCCCCGGGACGGCCGATGCCGCGTTA	LytCE
FlgMF	GTTAGGTACCGATCATTGTCTAGATTG	FlgM
FMNtR	CTGCTCGAGCATCTTCACCTCAATTTC	FlgM
FMCtF	CTGCTCGAGCAAGTAATGAGAGGGGGCTC	FlgM
FMCR2	CATCCCGGGTTGAGCGTCACCGTAAC	FlgM
ClpUp	GTAGGTACCCAGCAGGCTCTAAATGTTG	ClpP
ClpUpR	GTAGGATCCTCGAGAATAAATGTCATACG	ClpP
CDoF	GGATCCTCGATGAATGCGATAAGAG	ClpP
CDoR	GATATCTGTCTATCTTGTCCGCTTG	ClpP
LacF	CTAGGTACCAATCGCTCACATCTC	AprX
AprXR1	CGACTCGAGCTGTACCATGGAATATCC	AprX
AprXF2	CTACTCGAGCAGCAGAGGGTGCGATTC	AprX
AprXR2	CTACCCGGGGACGATTCCGAGATGAAGCAC	AprX
*σ^D^*Kpn	CTCGGTACCCTCGCGTTACGCTCTTTCTGT	FliC
FliCendR	CGACGGATCCGCACTCGAGACGAAGTAATTGTAATAC	FliC
FlagXhoF	GAGACTCGAGGAATCTGGAGCAGATTATA	FLAG tag
FuzendR2	GTCTAGGATCCTTAAAACCAATTCCATAATG	Enfuvirtide
NRev	CTTAGTCGACACCTGCAGCATCGTCTCC	N-terminal FliC
CFOR	CTACGTCGACCGCTCTTACCTAGGAGCT	C-terminal FliC
CstartR FuzF FuzR	GTCTGTCGACCATTAAAATTTCCTCCTTG GCTGTCGACATGTATACGTCATTAATTCATTCATTAATTGAAGAATCACAAAATCAACAAGAAAAAAATGAACAAGAATTATTAGAATTAGATAAATGGGCATCATT ATGGAATTGGTTTATGTGGATCCTCG CGAGGATCCACATAAACCAATTCCATAATGATGCCCATTTATCTAATTCTAATAATTCTTGTTCATTTTTTTCTTGTTGATTTTGTGATTCTTCAATTAAAGTATGAATTA ATGACGTATACATGTCGACAGC	C-terminal FliC Enfuvirtide Enfuvirtide

^a ^Restriction enzyme sites are underlined.

### Gene cassette construction

**Figure 1:**
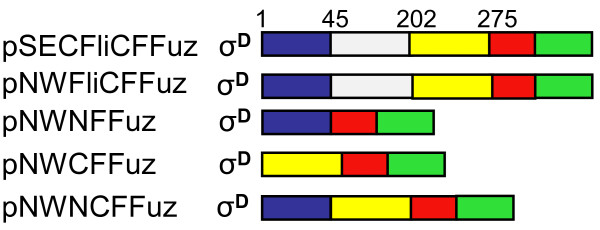
**Schematic representation of the flagellin fusion constructs containing different regions of the FliC protein fused to the FLAG tag and the Enfuvirtide peptide (Fuz) expressed in *B. halodurans *BhFDL05S**. The numbers indicate the size (amino acids) of the different fragments of the FliC protein used for the different constructs. The different colour blocks indicate the different regions of the FliC protein: the full-length flagellin protein (FliC_1-275_), the N-terminal region (FliC_1-45_) in blue, the C-terminal region (FliC_202-275_) in yellow and a truncated version with 157 amino acids of the central variable region (grey) deleted (FliC_Δ45-275_). The red block indicates a FLAG tag and the green block the Enfuvirtide (Fuz) peptide. An enterokinase cleavage site was inserted between the FLAG tag and Enfuvirtide peptide.

Synthetic oligonucleotides (FuzF and FuzR, Table 2) were derived from the Enfuvirtide amino acid sequence (YTSLIHSLIEESQNQQEKNEQELLELDKWASLWNWF) and codon usage optimised for *B. halodurans *[10]. The oligonucleotides were annealed according to the method described by Integrated DNA Technologies http://www.idtdna.com, digested with *Sal *1 and *Bam *H1 and ligated into pNW33N. In order to facilitate secretion, isolation and purification of the recombinant peptide, expression cassettes were developed as shown in Figure 1. Primers used to amplify the various fragments of the flagellin gene, the FLAG tag, the enterokinase cleavage site and the Enfuvirtide sequence are detailed in Table 2. All constructs were sequenced to confirm the desired DNA sequence and the correct reading frame.

### Western blotting and cell lysis determination

Samples were taken at different time points and the cell-free culture supernatants were analysed for the presence of fusion protein through SDS- PAGE and Western blots. Western blot analysis was carried out according to Gallagher et al [11] using Anti-FLAG M2 monoclonal antibody peroxidase conjugate from Sigma-Aldrich at a dilution of 1:2000 or rabbit polyclonal Anti-Enfuvirtide antibodies conjugated to peroxidase obtained from ANASPEC at a dilution of 1:1000. TMB (3,3',5,5'-Tetramethylbenzidine) solution (Sigma-Aldrich, Cat# T0565) was used for colour detection according to the manufacturer's specifications. As a control against the possibility that the heterologous fusion proteins in the growth medium could be a result of leakage or lysis from the cells, we analyzed intracellular and cell-free supernatant samples by Western blot analysis using a chloramphenicol acetyltransferase antibody (Abcam) at a dilution of 1:10 000 [4]. Intracellular samples were prepared as follows: cell cultures (5 ml) were centrifuged, the pellet resuspended in phosphate buffer (5 ml), sonicated for 3 × 5 min, centrifuged and 20 μl of supernatant evaluated on SDS-PAGE gel.

### Analysis of fusion protein concentrations

An ELISA plate assay using Anti-FLAG M2 monoclonal antibody peroxidase conjugate (1:5000) (Sigma A8592) was used to determine fusion protein concentrations. The procedure is based on direct adsorption of the fusion protein onto an ELISA plate. Cell free supernatant samples were used for analysis. The assay was essentially carried out according to the manufacturer's specifications (Sigma-Aldrich). For detection the substrate ABTS (2,2'-Azino-bis[3-ethylbenzo thiazoline-6-sulfonic acid]diammonium salt) (Sigma A1888) was dissolved (3 mg/10 ml) in citric acid (0.05 M, pH 4) and hydrogen peroxide (10 μl/10 ml) added just before use. An equal volume of 2% SDS was added to stop the reaction and the absorbance was read at 405 nm. The C-terminal FLAG-BAP™ control protein (Sigma P-7457) was used to create a standard curve. The amount of Enfuvirtide peptide present is calculated as a fraction of the total size of the fusion protein and is presented as mg Enfuvirtide per grams dry weight where OD_600 _1 = 0.35 g dry weight (gdw) [12].

Densitometric analysis of the Coomassie blue-stained SDS-polyacrylamide gels was also used to determine Enfuvirtide::flagellin fusion concentrations. Gel pictures were captured using a SynGene G-Box transilluminator and the GeneSnap version 7.01 software. Concentrations were determined using Gene Tools version 3.09 software. Bacterial alkaline phosphatase::FLAG protein (Sigma P-7457) at concentrations of 100, 200 and 400 ng was used to generate a standard curve. Peak areas of the relevant protein bands were used to calculate the protein concentration derived from the standard curve. This method allows for determination of the relative levels of the fusion proteins in the medium [6].

## Results

### Construction of *B. halodurans *BhFD05 mutant strains through gene-targeted inactivation

Modifications were made to the genome of *B. halodurans *BhFD05 to generate a number of strains with different genotypes with a view to evaluating their effect on the flagellin type III secretion pathway. The flagellum is composed of basal body proteins, proteins that form the external filament and hook proteins. Assembly of the flagellar organelle in *B. subtilis *is coupled to gene transcription and is governed by the flagellar-specific transcription factor σ^D ^and its antagonist, the anti-σ^D ^factor FlgM [13]. Thus, the genes chosen for inactivation included transcriptional regulators affecting σ^D^-dependent gene transcription, as well as proteases, specifically intracellular and ATP-dependent proteases, in an effort to further protect heterologous flagellin fusions with potential proteolytic susceptibility.

#### Construction of BhFDL05S

The *spo0A *and *lytCE *(lytic operon comprising the *lytC *and *lytE *genes) were inactivated on the chromosome of BhFD05, giving rise to strain BhFDL05S. The Spo0A protein is a master regulator of the entry of *Bacillus *sp. into sporulation [14]. It does, however, also have a pleiotropic effect on a number of different cellular processes, which include protease production [15], motility, competence for transformation [16] and biofilm formation [17]. Significant mutation of *spo0A *was found to result in abnormally high levels of σ^D^-dependent gene transcription [18]. As this is the sigma factor involved in *fliC *promoter activity, this gene was inactivated with a view to increasing flagellin expression. Kodama et al [19], however, reported that a *B. subtilis spo0A *mutant strain used as a host for extracellular protein production was found to have significantly low protease activities, but that these strains were also found to be more prone to cell lysis. This was attributed to decreases in the expression levels of extracellular proteases that led to stabilization of the autolysins. Autolysins are bacterial enzymes that hydrolyse and remodel the peptidoglycan found in the bacterial cell walls. In *B. subtilis *LytC, LytD and LytF have all been implicated in vegetative cell separation and cell motility [20]. In a strategy to prevent autolysis of the cells, Kodama et al [19] was able to show that inactivation of the major autolysin LytC was effective in preventing cell lysis of a *spo0A *mutant strain in *B. subtilis*. Consequently, both the *spo0A *gene and *lytCE *operon of *B. halodurans *was inactivated and their effect on the cells with regards to type III secretion, as well as cell lysis was evaluated.

#### Construction of BhFDL05SM

The *flgM *gene was deleted on the chromosome of BhFDL05SM, giving rise to strain BhFDL05SM. In enteric bacteria, the genes involved in flagellar synthesis and assembly can be grouped into three tiers. Class I includes the master genes *flhDC*, which are absolutely required for expression of class II genes, encoding the structural component of the hook-basal body (HBB) complex. The expression of class III genes encoding hook-associated proteins, flagellin, motor components and the chemotaxis apparatus is dependent upon completion of the HBB complex. Coupling of class III gene expression to HBB complex assembly is achieved through action of FlgM, an anti-sigma factor. In *B. subtilis *a similar hierarchy of genes involved in flagellar synthesis and assembly can be identified, even though no master operon corresponding to class I genes of enterobacteria has been described. By analogy to *E. coli *and *S. typhimurium*, genes involved in the HBB complex are grouped into class II, and genes whose expression is dependent on class II genes are considered to belong to class III. Class III genes constitute the σ^D ^regulon, since they are transcribed uniquely from promoters recognized by the σ^D ^initiation factor. The σ^D ^regulon includes genes involved in flagellar synthesis, motility, chemotaxis, autolysis and regulation of gene expression. Caramori et al [21] found that in *B. subtilis *FlgM is a major regulator of σ^D^-dependent promoters; in the absence of FlgM not only a strong increase in the expression of σ^D^-dependent promoters was observed, but also prolonged expression, protracted into the stationary phase. They speculated that FlgM may be involved in the temporal control of late flagellar gene expression. In order to determine whether this is the case in *B. halodurans *the *flgM *gene was deleted and its effect on recombinant peptide expression evaluated.

#### Construction of BhFDL06S and BhFDL05SC

The intracellular protease gene (*aprX*) and the ATP-dependent *clpP *protease gene of *B. halodurans *BhFDL05S were both deleted, giving rise to strains BhFDL06S and BhFDL05SC, respectively. Kodama et al [22] found that *aprX *was transcribed during stationary phase growth and the protease activity of AprX was detected in the culture medium of *B. subtilis *due to cell lysis. It was therefore decided to inactivate the *aprX *gene with a view to increasing the stability of the recombinant peptide in the extracellular medium.

In *B. subtilis*, Clp proteases are the main components of ATP-dependent degradation and Clp mutants show highly pleiotropic phenotypes. They are complexes in which the core is composed of the ClpP protein with low intrinsic proteolytic activity. In the complex with a hexameric ATPase, both high proteolytic activity and substrate specificity is conferred. No common sequence motifs could be determined for recognition by Clp proteases and Krűger et al [23] were able to show that Clp proteases participate directly in overall proteolysis of misfolded proteins. Pummi et al [24] investigated the role of Clp proteases in protein secretion and found that inactivation of ClpP improved the secretion of a heterologous protein by *B. subtilis*.

### Evaluation of different host strain backgrounds with regard to the flagellin-specific export pathway

**Figure 2:**
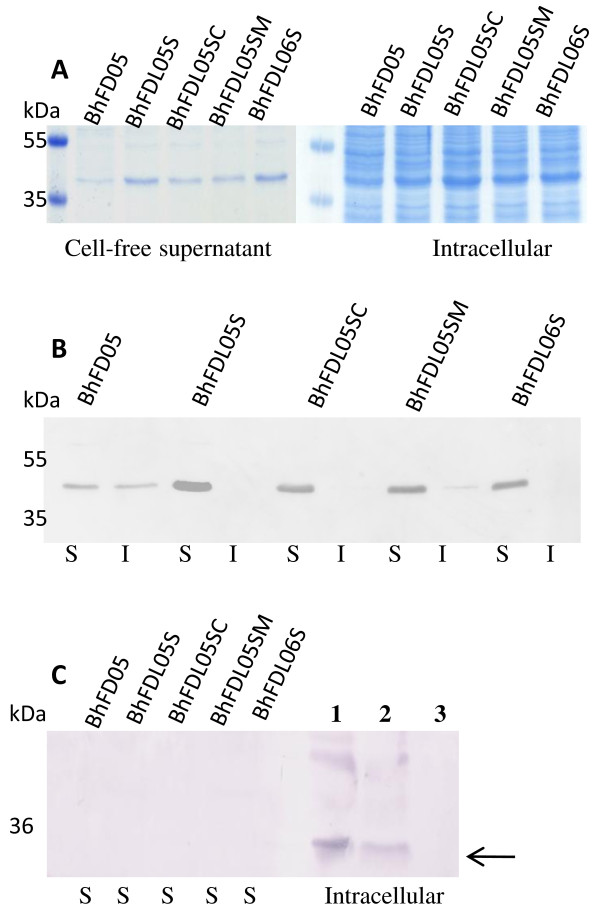
**SDS-PAGE and Western blot analysis of different fractions of *B. halodurans *recombinant strains transformed with plasmid pSECFliCFFuz**. **(A) **SDS-PAGE (12%) analysis of 16 h crude cell-culture supernatants (20 μl) and intracellular fractions (20 μl) from the different *B halodurans *strains as indicated above each lane. **(B) **Western blot analysis of the same samples as in A using Anti-FLAG monoclonal antibodies. The letter S indicates cell-free culture supernatant and I intracellular fraction. **(C) **Western blot analysis with an antibody to chloramphenicol acetyltransferase as a test for cell lysis. The crude cell-free supernatants (S) are the same as in A and the intracellular fractions are from control strain BhFDL05S (pSECFliCFFuz), lane 1; BhFDL05S (pSEC194), lane 2 and negative control BhFDL05S without plasmid, lane 3. The arrow indicates the expected size for chloramphenicol acetyltransferase (35 kDa). The molecular weight markers in kDa are indicated on the left.

**Figure 3:**
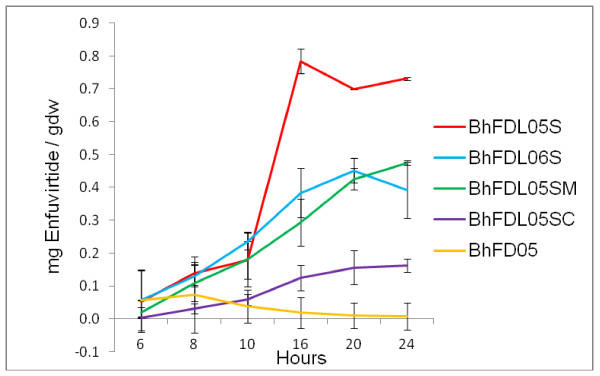
**Comparison of amount of Enfuvirtide peptide (mg/gdw) produced in the different *B. halodurans *strains containing pSECFliCFFuz**. An ELISA plate assay using Anti-FLAG antibodies was used to determine the concentration of fusion protein produced in the culture supernatants of the different strains at selected time points. The amount of Enfuvirtide peptide was calculated and presented as mg Enfuvirtide per grams dry weight. The error bars show the ± SD of three separate experiments.

The *B. halodurans *strains BhFDL05S, BhFDL05SC, BhFDL05SM and BhFDL06S were all transformed with pSECFliCFFuz (Table 1). These strains were evaluated in shake flask studies by comparing expression levels of recombinant flagellin fusion proteins secreted into the supernatants over a 24 h period. The strains were grown as described in the methods section. Cell-free culture supernatants and intracellular fractions were harvested at 16 h and 20 μl aliquots separated on an SDS-polyacrylamide gel (Figure 2A) and the fusion proteins were identified by Western blot (Figure 2B). In the publication of Berger et al (8) all the samples analysed on SDS-PAGE gels were concentrated cell-free supernatants, however in this study all samples evaluated were crude (non-concentrated) cell-free culture supernatants. The amount of flagellin fusion peptide secreted was analysed at various time points using an ELISA plate assay, as described in the Methods section, and actual Enfuvirtide peptide was calculated as mg of actual Enfuvirtide peptide per gram dry weight (mg/gdw) (Figure 3). From Figure 3 it can be seen that strain BhFDL05S with the *spo0A *gene and the lytic operon deleted showed the greatest improvement in the secretion of recombinant peptides, as compared to the parental strain BhFD05. This was followed by strains BhFDL05SM with the *flgM *gene deleted, and BhFDL06S with the intracellular protease gene *aprX *deleted. Strain BhFDL05SC with the *clpP *gene deleted showed the least improvement in peptide secretion.

The samples analysed in Figure 2A and 2B were evaluated by Western blot for the presence of chloramphenicol acetyltransferase in the cell-free supernatant fractions which would indicate cell lysis (Figure 2C). The marker protein was only detected in the intracellular fraction of the control strain BhFDL05S containing the plasmid pSEC194. This indicates that the heterologous fusion proteins were indeed secreted and that cell lysis did not occur during this time period. Thus, strain BhFDL05S was found to be the most efficient in secreting recombinant peptides via the flagellin type III secretion pathway and was therefore used as host strain for the evaluation of different constructs.

### Rationale for construct development

The export substrates of type III export systems do not contain cleavable signal sequences and they do not share common sequence motifs responsible for export. However, both Majander et al [4] and Végh et al [5] have identified different flagellum-specific secretion signals in two Gram-negative bacteria, *E. coli *and *Salmonella*, respectively, which they attribute towards mediating the secretion of flagellin through the flagellar export pathway. In *E. coli *this was identified as a 173-bp untranslated DNA fragment upstream of the *fliC *gene, and in *Salmonella *as a 22-residue long segment within the disordered N-terminal region of *Salmonella *flagellin. With this in mind, constructs were designed to contain the σ^D ^promoter region, full-length *B. halodurans *flagellin protein corresponding to amino acids 1-275 (FliC_1-275_), the N-terminal region (FliC_1-45_) of the, the C-terminal region (FliC_202-275_), and a truncated version of the flagellin protein with 157 amino acids of the central variable region deleted (FliC_Δ46-201_) (Figure 1). An *E. coli/Bacillus/Geobacillus *shuttle vector pNW33N was used to replace the pSEC194 vector, as it does not harbour a temperature sensitive *ori *of replication and was found to stably replicate in *B. halodurans *due to the presence of the cryptic replicon pBC1.

### Evaluation of the secretion efficiency of different constructs in *B. halodurans *strain BhFDL05S

**Figure 4:**
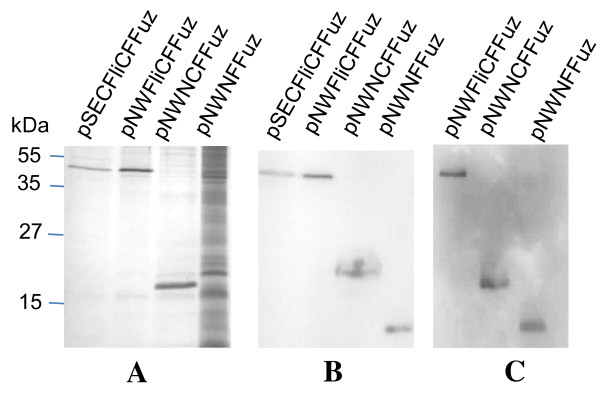
**SDS-PAGE and Western blot analysis of cell culture supernatants of *B. halodurans *BhFDL05S expressing different constructs**. (**A**) SDS-PAGE (15%) analysis of culture supernatants taken from 16 h cultures. The first three lanes each contained crude supernatant (20 μl) and lane 4 (pNWNFFuz) contained concentrated culture supernatant (equivalent to 250 μl of crude supernatant). This was due to relatively low concentrations of the fusion peptide. (**B**) Western blot analysis of same samples in (A) using Anti-FLAG monoclonal antibodies. (**C**) Western blot analysis of pNWFliCFFuz (38.2 k Da), pNWNCFFuz (19.8 k Da) and pNWNFFuz (10.5 k Da) with Anti-Enfuvirtide polyclonal antibodies. The amount of sample used for the Western blot was half of that used for the SDS-PAGE gel. Molecular weight markers in kDa are indicated on the left.

**Figure 5:**
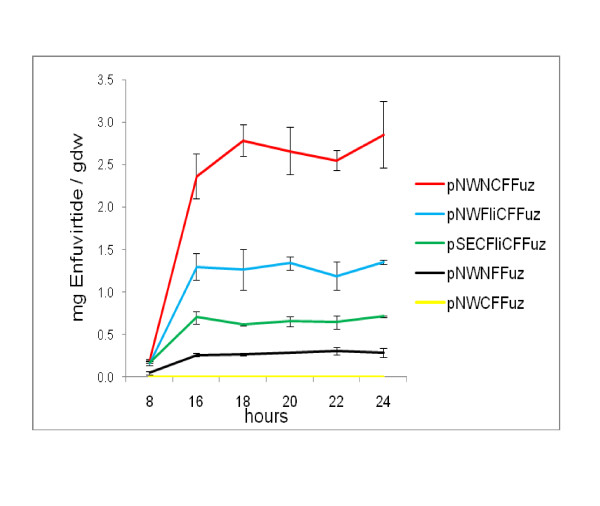
**Comparison of amount of Enfuvirtide peptide (mg/gdw) produced in *B. halodurans *BhFDL05S containing different expression cassettes**. The amount of Enfuvirtide peptide was calculated and presented as mg Enfuvirtide per grams dry weight. The error bars show the ± SD of three separate experiments.

All the constructs were transformed into strain BhFDL05S and were evaluated in shake flask studies by comparing expression levels of flagellin fusion proteins secreted into the supernatants over a 24 h period. Actual peptide concentrations were calculated to compensate for the size differences of the different peptide fusion constructs. Samples were taken and analysed as previously described. At 16 h, aliquots were separated on a SDS-PAGE gel (Figure 4A) and then analysed by Western blot (Figure 4B and 4C). From the results in Figure 5, it can be seen that the best construct in terms of peptide secretion is pNWNCFFuz, followed by pNWFliCFFuz and pSECFliCFFuz. The construct pNWNFFuz, containing only the N-terminal region of the flagellin protein, was found to secrete low levels of peptide into the medium, while for pNWCFFuz, containing only the C-terminal region, no recombinant peptide could be detected. The pNW33N vector replacement greatly benefitted expression, as did utilizing the truncated flagellin construct containing fragments of both the N- and C- terminal regions. Assays done on intracellular samples of BhFDL05S harbouring the different constructs showed very low levels of peptide intracellular (results not shown). The Coomassie blue-stained SDS-PAGE gel (Figure 4A) was subjected to densitometric analysis. The amount of actual Enfuvirtide peptide was calculated as a percentage of the total fusion protein (Table 3).

**Table 3:** Densitometric analysis of the fusion proteins from the Coomassie blue-stained SDS-PAGE gel (mg/L), as compared to ELISA results (mg/gdw)

Construct name	Fusion protein (mg/L)**	Enfuvirtide peptide (mg/L)*	Enfuvirtide peptide (mg/gdw)
**pSECFliCFFuz**	18.5	1.8	0.7
**pNWFliCFFuz**	32.9	3.3	1.3
**pNWNCFFuz**	61.0	13.4	2.8
**pNWNFFuz**	2.6	1.3	0.3
**pNWCFFuz**	ND	ND	0

*Enfuvirtide peptide calculated as a percentage of total fusion protein.

**Results quoted as the average of three experiments.

## Discussion

Most peptides are produced chemically by solid-phase and liquid phase synthesis, however, the longer the peptide the higher the cost of synthesis, as well as problems encountered with regard to chain aggregation. This leads to the formation of truncated peptides, which, in turn, impact on purification and yield. Microbial expression systems continue to be widely used in industry. Their intrinsic advantages such as rapid growth, high yields and ease of manipulation make them the premier choice for expression of non-glycosylated peptides and proteins [25]. We have developed an expression system focussed on the secretion of peptides and small proteins, which are vulnerable to proteolysis and therefore need to be expressed as protein fusions so as to enhance stability. In an effort to address the need for a cost-effective fermentation-based process for recombinant peptide production, we therefore evaluated the secretion of a therapeutic peptide, Enfuvirtide, by the *B. halodurans *flagellin type III secretion system through modification of both the host strain and expression cassette.

Initially, a number of different genetically engineered strains of *B. halodurans *BhFD05 were compared. The secretion efficiency of the fusion proteins was determined by comparing the levels of the recombinant peptide present in the medium, as determined by the ELISA assay. Our results clearly show that the BhFDL05S strain with both the *spo0A *and *lytCE *lytic operon deleted gave rise to the highest concentration of recombinant peptides in the supernatant. This was probably due to the increased levels of σ^D^-dependent gene transcription, as reported by Fawcett et al [18] to occur in *Bacillus*, as σ^D ^is the major sigma factor involved in *hag *gene transcription encoding not only the flagellin protein but also genes encoding proteins for flagellar synthesis, motility and chemotaxis [26]. The inactivation of the lytic operon was also found to enhance the stability of the cells and prevent cell lysis, as the intracellular protein marker, chloramphenicol acetyltransferase, could not be detected in the supernatant after 24 h of growth when evaluated by Western blot. The inactivation of *flgM *on the chromosome of strain *B. halodurans *BhFDL05S did not lead to a further increase in protein production. This could possibly be due to the pleiotropic effect of the Δ*spo0A *background. This would, however, need to be confirmed by transcriptomic and proteomic results. In contrast, inactivation of the intracellular protease *aprX *(strain BhFDL06) and the *clpP *protease (strain BhFDL05SC) did not play a significant role in improving recombinant protein levels.

A number of constructs were compared for their ability to secrete the recombinant peptide fusions using the modified type III flagellin pathway of strain BhFDL05S. Previous studies with regard to Gram-negative bacteria suggested that the signal sequence is found at the mRNA level [27,4] or that a combination of an N-terminal signal and a structural element in the mRNA is required [28]. Alternatively, the signal is contained at the protein level and is located at or near the N terminus [5,29-32]. In contrast to these findings in Gram-negative bacteria, we found that a construct encoding a truncated flagellin gene fused to the Enfuvirtide peptide with both the N-terminal and C-terminal regions present was best able to mediate the secretion of fusion peptide into the supernatant. The N-terminal region contained the 22-residue long segment identified by Végh et al [5] as containing the recognition signal for the flagellar export machinery. In this study, all the constructs also contained 230 bp of untranslated sequence upstream of the *fliC *gene in the σ^D^-promoter region, which Majander et al [4] claimed facilitated the extracellular secretion of polypeptides via the type III pathway in *E. coli*.

Low levels of peptide could be detected in the supernatant with the construct containing only the N-terminal region (pNWNFFuz) and none could be detected with the construct containing only the C-terminal region (pNWCFFuz). Intracellular levels were extremely low for constructs pNWFliCFFuz and pNWNCFFuz and none for pNWNFFuz and pNWCFFuz (results not shown). Majander et al [4] reported that N-terminal polypeptides lacking the C-terminus were efficiently secreted when the corresponding gene fragments were expressed from the *fliC *promoter, but remained within the cell when non-natural promoters were used. However, we found that in *B. halodurans *a combination of both N- and C-terminal regions are necessary in order to be able to successfully secrete increased levels of flagellin-peptide fusions. With the C-terminal region alone, no flagellin fusion peptide was observed, either intra- or extracellularly, indicating that the C-terminal fusion is unstable. However, it has been proposed that in the Gram-negative flagellar system the C-terminal region of the flagellin protein is involved in the chaperone binding, therefore playing a role in the stabilization of the flagellin monomers from proteolytic activity, as well as in prevention of premature folding and in the timing of secretion of the flagellin monomers from the cytoplasm [33,34].

A major advantage of secreted protein expression is that cell disruption is no longer required before purification and the starting material is less contaminated with host proteins, lipopolysaccharides and nucleic acids. The constructs contain a FLAG-tag that enables easy detection and purification, and an enterokinase cleavage that facilitates selective removal of the peptide of interest. Our system utilizes an endogenous promoter and therefore no inducers need to be added to the medium. Yields of actual peptide were calculated from peptide fusions in the supernatant using the ELISA assay, which allows for a comparative determination of peptide yields between the different constructs. Strain optimization increased actual peptide levels by almost 10-fold and cassette optimization contributed a further 3.5-fold increase. In addition, we evaluated peptide fusion concentrations using densitometric analysis that was also used to determine secretion levels obtained from either the *Salmonella *or *E. coli *flagellar type III systems. Since the amount of Coomassie blue stain bound to the same amounts of a range of proteins might differ, this method is not directly comparable. It is, however, of interest to note that with the *E. coli *secretion system peptide fusions of between 1 and 15 mg/L were obtained in the supernatant. However, the Peb1 protein, which was found to be secreted at the highest levels, was also recently found to be actively secreted by *E. coli *in a flagellar-independent manner by an unknown two-step process [35]. In the *Salmonella *system, a range of different protein fusions, eighteen in total, were found to be secreted at levels varying between < 1 and 4 mg/L. A number of these were found to form intracellular aggregates. From densitometric analysis of recombinant flagellin fusions secreted by *B. halodurans*, concentrations of 61 mg/L of peptide fusions were obtained after 16 h using strain BhFDL05S expressing the pNWNCFFuz construct. This translates into approximately 13 mg/L of actual peptide being secreted as a fusion in shake flask cultures. No intracellular aggregates were identified with this construct. Cleavage by enterokinase is designed to yield the authentic amino-terminus of the peptide as no additional amino acids remain on the N-terminal region of the peptide after cleavage, thereby allowing for the chemical modification step to follow. This is a promising result for biotechnological applications, as there is clearly potential for further optimization through fermentation studies.

## Conclusions

The type III flagellar secretion system of *B. halodurans *has been shown to successfully secrete a therapeutic peptide as a flagellin fusion polypeptide, giving rise to possibilities for biotechnological applications using this novel engineered production host. Our results demonstrate that chromosomal inactivation of both the *spo0A *gene and the lytic operon increased the levels of the secreted peptide fusions. In addition, further improvement in the concentration of the secreted fusion peptides was achieved by the incorporation of both the N-terminal and C-terminal flagellin regions of the flagellin monomer in structuring an optimized cassette on a multi-copy vector. In shake flask studies, strain BhFDL05S expressing the pNWNCFFuz construct, was shown to secrete 61 mg/L of peptide fusions into the supernatant after 16 h, which translates into approximately 13 mg/L of actual Enfuvirtide peptide.

## Competing interests

The authors declare that they have no competing interests.

## Authors' contributions

EB participated in the design of the study, carried out gene targeted inactivation, evaluation of mutant strains by ELISA and helped in drafting the manuscript. NN participated in the molecular genetic studies. MC participated in the design of the study, development of the Enfuvirtide construct and carried out the Western blots. ML had the main responsibility for the design and coordination of the project and drafted the manuscript. All authors read and approved the final manuscript.
